# Supplementary material for: MetaRibo-Seq measures translation in microbiomes
Source: Nat Commun. 2020 Jun 29;11:3268. doi: 10.1038/s41467-020-17081-z (PMC7324362; doi:10.1038/s41467-020-17081-z)
Supplement: Supplementary file 10 — Supplementary Data 7 [file 41467_2020_17081_MOESM10_ESM.zip › File2/Confidence_High_Taxonomy/16767_out.krona.html]

Javascript must be enabled to view this page.

members
magnitude
magnitudeUnassigned
count
unassigned
taxon
rank

16767\_out

2

2
superkingdom
2

2
phylum
1239

2
class
186801

order
186802
2

186803

SRS074670\_contig\_number\_contig-100\_32207.32207
1
family
2

189330
genus
1

SRS013965\_contig\_number\_contig-100\_41807.88792
